# Traumatic renal artery thrombosis: a case report and literature review

**DOI:** 10.3389/fmed.2024.1480275

**Published:** 2024-12-09

**Authors:** Xiaohua Xia, Guang Zhao, Qiupeng Feng, Zhiqiang Guo, Jin Ma, Hua Yuan

**Affiliations:** Department of Emergency Medicine, The First People’s Hospital of Kunshan, Kunshan, China

**Keywords:** trauma, traumatic renal arterial thrombosis, diagnosis, therapy, case report

## Abstract

Traumatic Renal Artery Thrombosis (TRAT) is an uncommon condition characterized by a low clinical incidence and a lack of specific symptoms, making it highly susceptible to being overlooked or misdiagnosed. This retrospective analysis aims to review the diagnostic and therapeutic approaches applied to a TRAT patient who received successful treatment in the Emergency Medicine Department of the First People’s Hospital of Kunshan. The objective is to enhance clinicians’ comprehension of TRAT, promote early diagnosis and intervention, and ultimately improve patient outcomes.

## Introduction

Traumatic Renal Artery Thrombosis (TRAT), also referred to as traumatic renal autotomy, traumatic renal infarction, or traumatic renal artery occlusion, constitutes only 1–4% of renal artery injuries resulting from abdominal trauma (1, 2). TRAT is a rare condition that predominantly affects the left renal artery, with bilateral occurrences being exceptionally rare. The clinical presentation of TRAT is often subtle and nonspecific, which contributes to its frequent misdiagnosis or overlooked diagnosis. Previous research has primarily concentrated on patients with underlying internal diseases, with traumatic renal artery thrombosis largely documented through isolated case reports. In this report, we present a case of TRAT that was successfully managed in the Emergency Medicine Department of the First People’s Hospital of Kansan.

## Case presentation

An 18-year-old female patient was admitted to our emergency department at 17:33 following a traffic accident that resulted in 1 h of unconsciousness. On physical examination, she was in a moderate coma with the following vital signs: temperature 36.5°C, heart rate 123 beats per minute, blood pressure 58/38 mmHg, and SpO2 96%. Her Glasgow Coma Scale score was E1V1M2. Her pupils were 2 mm in diameter bilaterally with a slow response to light. Examination revealed multiple contusions, wet lung sounds bilaterally, regular breathing patterns, and a soft abdomen.

**Figure 1:**
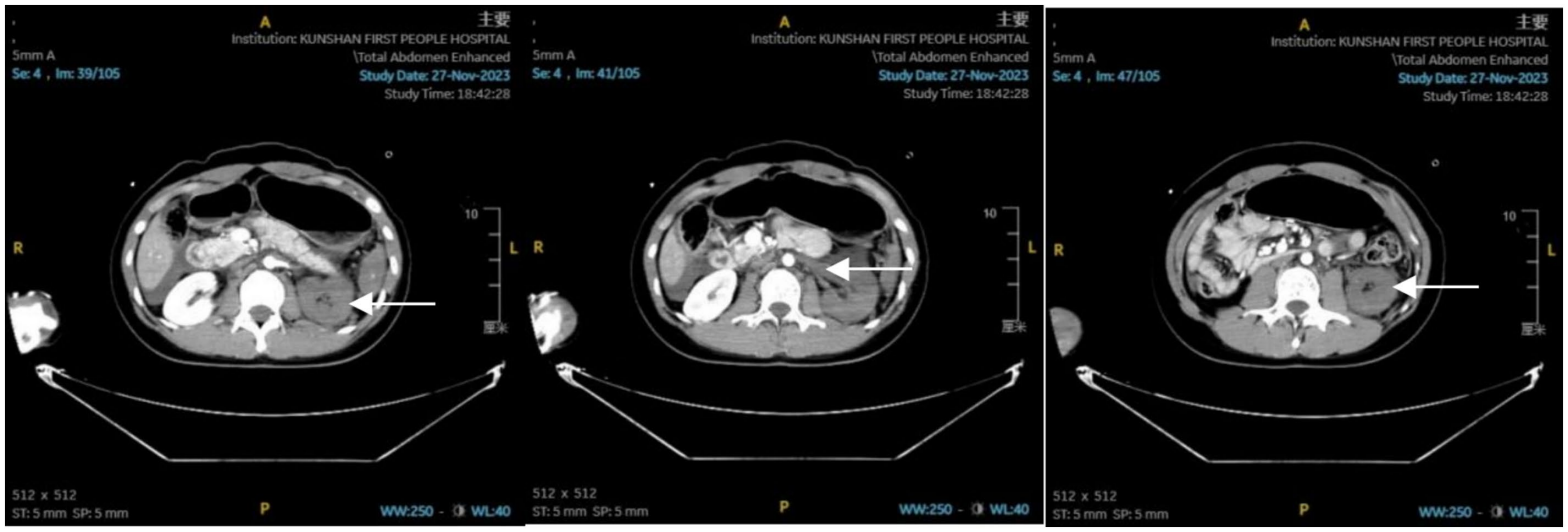
Abdominal enhanced CT report: contusion of the left hepatic lobe; contusion of the spleen; contusion of the left adrenal gland; embolism of the left renal artery; presence of abdominal and pelvic effusion.

Immediately after admission, the patient was stabilized with cervical collar, chest, and pelvic fixation. Endotracheal intubation was performed for assisted ventilation, and deep venous catheterization was carried out. A bedside ultrasound eFAST examination was conducted, revealing perisplenic and abdominopelvic effusions. Fluid resuscitation included 400 mL of plasma, 4 units of MAP (suspended leukocyte-reduced red blood cells), and 1,500 mL of crystalloid solution. Norepinephrine was administered to maintain the mean arterial pressure (MAP) around 65 mmHg. Initial blood tests: WBC: 9.95 × 10^9^/L, Hb: 118 g/L, PLT: 170 × 10^9^/L; BUN: 8.51 mmol/L, Scr: 65.6 μmol/L; Blood gas analysis: pH: 7.32, lactate: 3.5 mmol/L, base excess: −6.0 mmol/L. Whole-body CT at 18:27 revealed: No obvious traumatic changes in the brain or cervical spine; Bilateral lung contusion, especially on the left side; Small bilateral pneumothorax; Fractures of the sternal stem, left scapula, T1, T4–T7 right transverse process, right 6th posterior rib, T4 and T5 burst fractures, T6 and T7 fractures; Spleen contusion, left adrenal contusion, abdominal and pelvic effusion. Enhanced abdominal CT (Figure 1) at 20:05 revealed: Left hepatic lobe contusion and laceration; Spleen contusion and laceration; Left renal artery embolism; Left adrenal gland contusion; Massive abdominal and pelvic effusion.

At 21:10, the patient was sedated and transferred to the operating room for a splenectomy, with a temperature of 36.3°C, pulse of 114 bpm, BP of 117/68 mmHg, and SpO2 of 100%. The patient was transferred to the EICU at 23:55 and continued to receive fluid resuscitation, analgesia and sedation, anti-infection treatment, stress ulcer prevention, organ function protection, nutritional support, and other necessary treatments. During the night, 400 mL of plasma and 2 units of MAP were infused, bringing the total fluid intake to 3,565 mL.

The next day’s blood test results: WBC: 25.38 × 10^9^/L, Hb: 110 g/L, PLT: 76 × 10^9^/L; ALT: 167 U/L, AST: 330 U/L, LDH: 1,693 U/L, Albumin: 26.3 g/L, BUN: 7.99 mmol/L, Creatinine: 117.7 μmol/L; Coagulation index: APTT: 35.6 s, D-dimer: 29.35 mg/L; Blood gas analysis: pH: 7.4, Lactate: 2.2 mmol/L, Base excess: −0.5 mmol/L. On the third day, CT and contrast-enhanced ultrasound showed an acceleration time of 180 ms in the left renal artery, with a blood flow spectrum indicating “delayed time to peak,” suggestive of severe stenosis or occlusion. On the sixth day, based on CT results and pathogen identification, anti-infective therapy was intensified with piperacillin-tazobactam and linezolid. On the 12th day, B-ultrasound indicated no abnormalities in the bilateral renal arteries. Biochemical tests showed BUN: 7 mmol/L and Scr: 77.4 μmol/L. On the 16th day, the patient underwent orthopedic robot-assisted open reduction and internal fixation for a thoracic vertebral fracture. On the 17th day, the patient was successfully extubated. Biochemical tests showed BUN trending down to 6.29 mmol/L and Scr: 66.7 μmol/L.

**Figure 2:**
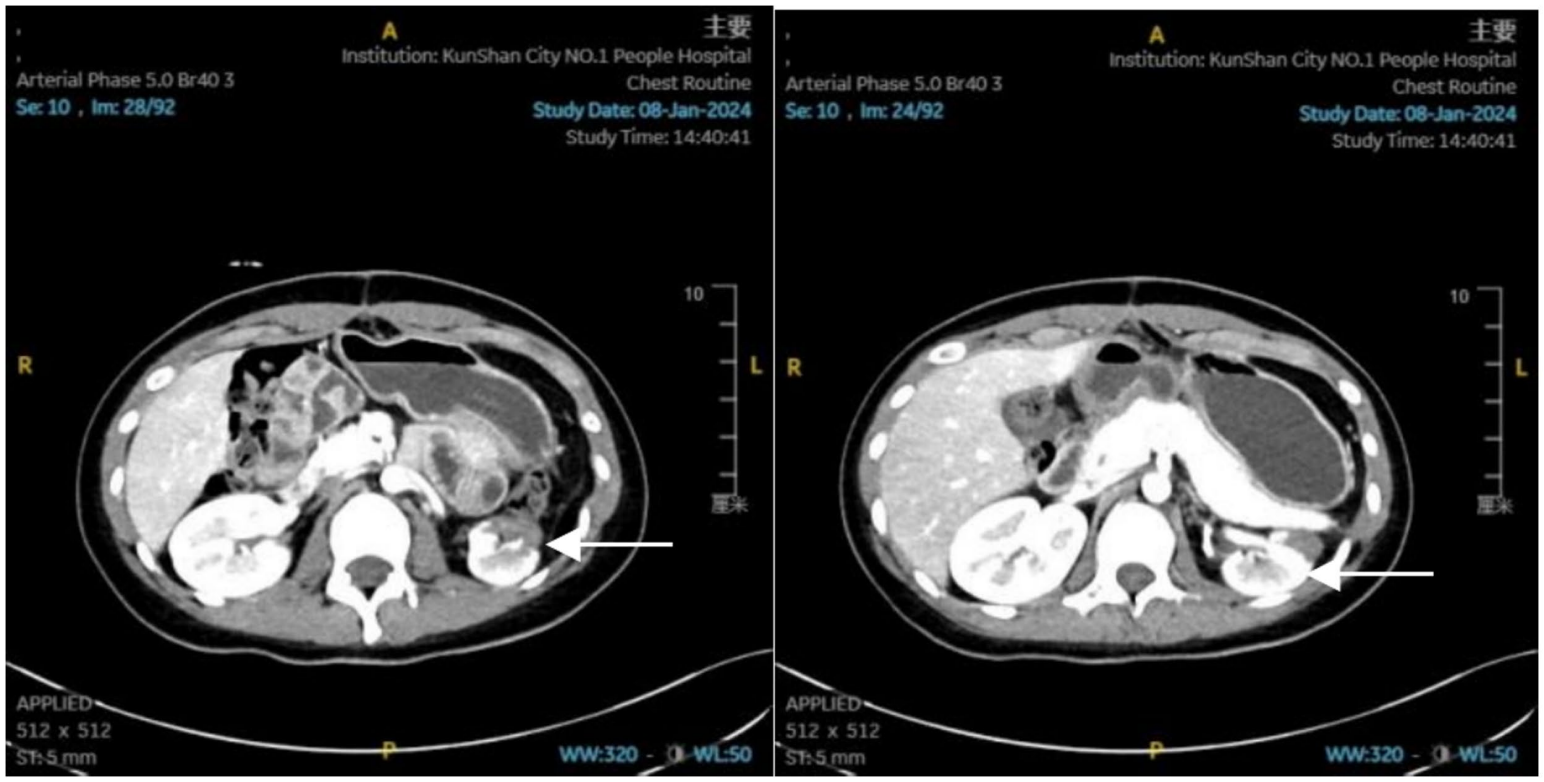
Abdominal enhanced CT report: status post-splenectomy; contusion of the left lobe of the liver, which has shown significant improvement. Contusion of the left adrenal gland, also showing significant improvement. Slight atrophy of the left kidney.

By the 22nd day, the patient’s condition had sufficiently improved to be transferred to a general ward. Biochemical review showed BUN trending up to 7.0 mmol/L and Scr: 77.4 μmol/L. Before discharge, an enhanced CT scan (Figure 2) revealed post-splenectomy improvements with contusions in the left lobe of the liver and left adrenal gland. The left kidney showed slight atrophy, which also demonstrated improvement. The patient was discharged one and a half months later.

## Discussion

In a large study of 945,326 trauma patients, the incidence of renal artery injury was found to be 0.05% (3). TRAT was even rarer and frequently associated with other abdominal injuries. The mechanism of TRAT is generally attributed to sudden acceleration or deceleration of the body, causing sharp movement of the kidney. This generates significant force on the renal pedicle. While the vascular adventitia and muscle layer can stretch due to their elasticity, the endothelial lining lacks this flexibility and consequently suffers varying degrees of trauma or laceration. Following endothelial injury, thrombosis forms due to hemodynamic changes, gradually leading to renal artery occlusion, decreased renal blood flow, and eventually renal dysfunction, ischemia, and necrosis (4, 5). Due to the subtle nature of symptoms and the limited reports on TRAT in the literature, clinicians often lack sufficient understanding and emphasis on the condition. This can easily result in missed or incorrect diagnoses. Following TRAT, symptoms such as abdominal pain, flank pain, and hematuria may occur, though they are non-specific. The clinical manifestations depend on the size of the affected blood vessels and the extent and scope of the infarction. Additionally, patients with TRAT often have concurrent injuries to other parts of the body, which can obscure the clinical symptoms.

Renal CT plain scan and enhanced scan combined are considered to have a diagnostic rate as high as 98%. However, in clinical practice, we have observed that a CT plain scan alone may not sufficiently reveal early renal ischemia and detailed renal vascular injuries, leading to a high rate of missed diagnoses. In this case, an early abdominal enhanced CT scan facilitated the early detection and treatment of lesions, resulting in a favorable prognosis. Therefore, we recommend early abdominal enhanced CT scans to improve the early diagnosis rate, as supported by related reports confirming that abdominal enhanced CT is the preferred diagnostic method (5, 6). For TRAT patients, especially those with injuries involving multiple body parts, we recommend performing a whole-body enhanced CT scan as soon as possible (7). This approach minimizes the risk of missed diagnoses related to injuries across various anatomical regions. However, in some cases, patients may initially present with no obvious abnormalities on early CT examinations, only to develop symptoms of renal ischemia and other complications several hours or even days later. For instance, Jiansong et al. (8). reported a case of delayed renal infarction, while other studies have documented imaging changes appearing as early as 1.5 h post-injury (9). This situation demands vigilant attention and proactive monitoring to promptly identify and address any delayed complications.

Ultrasound plays a crucial role in the diagnosis and treatment of patients with TRAT, particularly for those with severe trauma who are unable to undergo early CT examination. Early bedside ultrasound serves as a valuable screening tool, providing critical information for physicians and facilitating prompt decision-making. Bedside ultrasonography, being noninvasive and convenient, can be utilized throughout the treatment process for continuous monitoring and evaluation. It enables ‘organ-level’ monitoring of the kidneys, delivering vital information for effective clinical management. Additionally, contrast-enhanced ultrasound is effective in monitoring and evaluating renal vascular conditions and kidney ischemia, offering detailed insights that aid in diagnosis and treatment. We believe that fully valuing and utilizing ultrasound examination in clinical diagnosis and treatment can significantly reduce patient transport risks, minimize radiation exposure, and lower medical costs.

Renal angiography is valuable for patients with TRAT detected by contrast-enhanced CT, especially when revascularization is planned (6). Voiding urography serves as an auxiliary diagnostic method to indicate changes in renal function and provide a basis for revascularization treatment, although it does not assist in detecting thrombosis. Isotope renal scanning reveals the position, shape, size, and function of the kidney, making it a valuable option for both diagnosis and follow-up (4).

In this case, despite changes in laboratory tests such as elevations, achieving a definitive diagnosis was challenging without the aid of imaging examinations. Continuous monitoring of renal function indices, such as creatinine and urea nitrogen, is essential during the treatment process. These indices may show varying degrees of change but lack high specificity. However, they can support decisions regarding blood purification and renal resection treatments. For patients with TRAT, ongoing follow-up of renal function indices and imaging should continue for at least 1 year (Table 1).

**Table 1:** Laboratory variables.

Laboratory variables	First day	Second day	12th day	17th day	22nd day
BUN (mmol/L)	8.51	7.99	7.0	6.29	7.0
Scr (μmol/L)	65.6	117.7	77.4	66.7	77.4
WBC (*10^9^/L)	9.95	25.38			
Hb (g/L)	118	110			
PLT (*10^9^/L)	170	76			
pH	7.32	7.4			
Lactate (mmol/L)	3.5	2.2			
BE (mmol/L)	−6.0	−0.5			

BUN, blood urea nitrogen; Scr, serum creatinine; WBC, white blood cell; Hb, hemoglobin; PLT, blood platelet; BE, base excess.

Currently, there is no clearly defined optimal treatment for TRAT. Traditionally, early nephrectomy has been performed to prevent adverse effects such as renal hypertension caused by an ischemic kidney. However, the current preferred approach is to attempt kidney preservation through careful observation and monitoring. This patient represents a successful case of kidney preservation achieved through continuous monitoring and observation during treatment.

### Non-revascularization

According to Jawas et al. (10), patients with unilateral renal artery thrombosis can be managed conservatively. They monitored the renal function of five patients treated conservatively, with three out of five patients followed for 9 months showing normal renal function indices. One case required renal resection due to hypertensive kidney disease, and another patient underwent nephrectomy due to hemodynamic instability. Singh et al. (1) reported a case of TRAT in an 8-year-old child who showed improvement with conservative treatment. Currently, most experts believe that non-surgical treatment is appropriate for patients with unilateral lesions, provided that blood pressure is closely monitored. Surgical intervention should be considered if renal hypertension cannot be controlled with medication (2). The reasons for recommending conservative treatment are as follows: (1) studies have shown that the success rate of revascularization is only 14–29%; (2) some studies have found that, due to late compensatory collateral circulation formation, renal function and blood flow can recover in some patients within a few weeks (11, 12); (3) in TRAT patients, thrombosis often involves both the trunk and peripheral vessels, making revascularization very difficult to achieve (13).

### Revascularization

Haas et al. (14) conducted a retrospective analysis of 12 patients with TRAT over a 15-year period and found that the success rate of revascularization was low. They concluded that revascularization should be considered only for patients with a renal ischemia time of less than 5 h and hemodynamic stability. For patients with an isolated kidney or bilateral RAT, vascular reconstruction should be promptly considered. If vascular reconstruction fails, Kidney auto transplantation should be considered. However, there is no consensus on the timing of TRAT revascularization. Some experts believe that renal tissue viability is maintained for approximately 24 h after the onset of renal ischemia, advocating for revascularization within this 24-h window or ideally within 2–4 h (2, 6). While some scholars argue that this timeframe may not be entirely accurate since the onset of renal ischemia does not necessarily coincide with the time of injury (15). Therefore, they caution against using the injury time as the sole reference standard for revascularization timing.

In conclusion, the treatment of TRAT should be evaluated to balance the benefits of restoring renal function against the potential risks, including increased mortality. The traditional method of revascularization involves renal revascularization, which can be performed using autologous or artificial blood vessel transplantation (11). Autotransplantation can be performed using the great saphenous vein or the splenic artery for anastomosis. With the rapid development of interventional medicine in recent years, endovascular stent revascularization therapy, also known as endovascular exclusion, has been increasingly chosen due to its minimal trauma and few complications. The procedure typically involves inserting a catheter through percutaneous puncture, aspirating the thrombus, placing an endovascular stent, and finally performing angiography to check for patency (2, 6). Lopera et al. (16), based on a study of interventional therapy for RAT in seven patients, suggested that renal artery stenting was successful in most cases. However, they noted an underlying factor of renovascular hypertension, which may later necessitate nephrectomy. When TRAT occurs, renal vascular injury can produce increased levels of angiotensin, leading to high blood pressure. The incidence of late-onset hypertension ranges from 15 to 57%, and it can develop over months or years. If hypertension occurs and conservative treatment is ineffective, nephrectomy may become necessary. Negoro et al. (9) reported a case of traumatic renal artery thrombosis resulting in renovascular hypertension, with elevated levels of renin, angiotensin I, and angiotensin II. The patient’s blood pressure normalized after nephrectomy. However, nephrectomy should not be performed prematurely in the absence of complications such as hypertension.

### Other treatments

Surgical thrombectomy is rarely employed due to its low success rate. Gao Jinmou reported three cases of thrombectomy; however, each case experienced recurrence within 24 h, resulting in renal ischemia (15). Additionally, there are clinical reports related to thrombolytic therapy. Nakayama et al. (17) treated a patient with TRAT using urokinase injections and systemic anticoagulation. A follow-up at 3 months revealed no renal artery occlusion. At the 15-month check-up, the diseased kidney had 50% of the function compared to the healthy contralateral kidney. Nevertheless, due to the lack of extensive case studies supporting thrombolytic therapy, we believe that TRAT patients typically present with bleeding or clotting disorders, leading to many contraindications for anticoagulation and thrombolysis. Furthermore, TRAT differs from renal artery thrombosis caused by internal medical reasons; late recurrence is still difficult to avoid due to the presence of intimal damage.

For trauma patients, especially those with abdominal injuries, it is important to be alert for the occurrence of TRAT. With the standardization of trauma treatment and the early application of enhanced abdominal CT, more cases of TRAT are being identified. Consequently, TRAT is now better recognized, allowing for earlier diagnosis and treatment. Current treatment tends to focus on close observation and monitoring to protect the kidney. However, revascularization should be considered in patients with a solitary kidney or bilateral RAT. With advancements in interventional medicine, TRAT patients now have the opportunity to restore renal blood flow and maintain renal function.

## Conclusion

In summary, we should prioritize the principle of ‘saving lives first, organ preservation second’. Additionally, it is crucial to enhance our understanding of TRAT, ensure early diagnosis and treatment, and thereby improve patient outcomes.

## Data Availability

The original contributions presented in the study are included in the article/supplementary material, further inquiries can be directed to the corresponding author.
